# Baumol’s cost disease in acute versus long-term care: Do the differences loom large?

**DOI:** 10.1007/s10754-025-09392-9

**Published:** 2025-02-24

**Authors:** Kaan Celebi, Jochen Hartwig, Anna Pauliina Sandqvist

**Affiliations:** 1 Faculty of Economics and Business Administration, Chemnitz University of Technology, Chemnitz, Germany; 2 KOF Swiss Economic Institute, ETH Zurich, Zurich, Switzerland; 3 Forum for Macroeconomics and Macroeconomic Policies, Hans Böckler Stiftung, Düsseldorf, Germany; 4 Deloitte GmbH Wirtschaftsprüfungsgesellschaft, Munich, Germany

**Keywords:** Health care expenditure, Baumol’s cost disease, Extreme Bounds Analysis, MM estimator, OECD panel, C12, C23, I10

## Abstract

Baumol’s (Am Econ Rev 57: 415–426, 1967) model of ‘unbalanced growth’ yields a supply-side explanation for the ‘cost explosion’ in health care. Applying a testing strategy suggested by Hartwig (J Health Econ 27: 603–623, 2008), a sprawling literature affirms that the ‘Baumol effect’ has both a statistically and economically significant impact on health care expenditure growth. Skeptics maintain, however, that the proliferation of hi-tech medicine in acute care is clearly at odds with the assumption underlying Baumol’s model that productivity-enhancing machinery and equipment is only installed in the ‘progressive’ (i.e. manufacturing) sector of the economy. They argue that Baumol’s cost disease may affect long-term care, but not acute care. Our aim in this paper is to test whether Baumol’s cost disease affects long-term care and acute care differently. Our testing strategy consists in combining Extreme Bounds Analysis (EBA) with an outlier-robust MM estimator. Using panel data for 23 OECD countries, our results provide robust and statistically significant evidence that expenditures on both acute care and long-term care are driven by Baumol’s cost disease, even though the effect on long-term care expenditures is more pronounced.

## Introduction

Baumol’s (1967) model of ‘unbalanced growth’ yields a supply-side explanation for the ‘cost explosion’ in health care. Baumol divides the economy into two parts: a ‘progressive’ and a ‘non-progressive’ sector. He assumes that productivity growth is higher in the progressive (secondary) than in the non-progressive—or ‘stagnant’—(tertiary) sector of the economy, but wages grow more or less the same in both sectors. Therefore, unit costs and also prices rise much faster in the tertiary sector than in the secondary. Demand for certain services, like health care and education for instance, is hardly price-elastic, hence consumers are willing to pay the higher prices. Therefore, even if the two sectors keep their proportion in terms of real production, an ever-higher share of total expenditures will be channeled into the stagnant sector. This phenomenon is known as ‘Baumol’s cost disease’.^1^

Hartwig (2008) has suggested a test of whether Baumol’s cost disease drives health care expenditure (HCE) in OECD countries that does not require price or productivity data for the health sector, which are notoriously unreliable (see Berndt et al., 2000, p. 171). This test consists in regressing HCE growth rates (log differences) on the difference between nominal wage growth and labor productivity growth in the overall economy (plus controls). Rossen and Faroque (2016, p. 192) neatly summarize the intuition behind this approach as follows: “His [Hartwig’s] key insight is that since wage growth in health care depends on the higher productivity growth in the rest of the economy, growth in the unit labor cost and price of health care services, and therefore growth in health care spending, must bear a proportional relationship to the excess wage growth over labor productivity growth in the overall economy”.^2^

Evidently, Hartwig’s ‘Baumol variable’, i.e. the difference between nominal wage growth and labor productivity growth, equals the growth rate of aggregate (nominal) unit labor cost (NULC). To check whether the ‘Baumol variable’ is not just picking up purely monetary changes, Hartwig (2008) deflated both per-capita HCE— the dependent variable—and nominal wages per employee on the right-hand side of the regression equation by the GDP deflator. Hence, his ultimate test for whether Baumol’s cost disease drives health care expenditure consists in regressing real HCE growth on the growth rate of aggregate real unit labor cost (RULC) plus controls.^3^ Hartwig (2008) and scholars following his lead to testing Baumol’s cost disease in health care (see, e.g., Bates & Santerre, 2013, Medeiros & Schwierz, 2013, Hartwig & Sturm, 2014, Rossen & Faroque, 2016, Colombier, 2017, Tian et al., 2018, Bellido et al., 2019, Lorenzoni et al., 2019, Jeetoo, 2020, Wang & Chen, 2021) have thoroughly confirmed that the ‘Baumol effect’^4^ has both a statistically and economically significant impact on health care expenditure growth.^5^

Even though empirical research over the past decade and a half has built up a strong case in favor of Baumol’s cost disease being one of the main drivers of HCE growth, there remains one piece of skepticism that motivates our present paper. This skepticism was first brought to our attention by the late Gebhard Kirchgässner, then president of the Swiss federal *Commission for Business Cycle Affairs* (KfK). The 2006 annual report of that commission titled ‘Reforming the health system’ (Kommission für Konjunkturfragen, 2006, pp. 36–37) quotes the working paper version of Hartwig (2008) rather disapprovingly. Technological progress, so the argument goes, is rife in acute care, becoming manifest in hi-tech medicine. Therefore, the assumption underlying Baumol’s model of ‘unbalanced growth’ that productivity-enhancing machinery and equipment is only installed in the secondary sector of the economy is clearly flawed. The report does concede an impact of Baumol’s cost disease on HCE growth, but restricts it to the long-term care (LTC) sector (see also Kirchgässner, 2009).

Similarly, de la Maisonneuve and Oliveira Martins (2014), in their health expenditure projections until 2060 on behalf of the OECD, model (public) HCE and LTC expenditure separately and allow Baumol’s cost disease (for which they use the level or the growth rate of labor productivity in the total economy as a proxy) only to affect the latter. In their update of the OECD’s spending projections, however, Lorenzoni et al. (2019, p. 25), note that the allowance of “the impact of the Baumol effect on health care as a whole (instead of only for long-term care)” was one of the main differences against previous studies.

Our aim in this paper is to test whether Baumol’s cost disease affects long-term care and acute care differently. Acute care, according to the OECD (2008, p. 17) “is one in which the principal intent is one or more of the following: (i) to manage labour (obstetrics), (ii) to cure illness or to provide definitive treatment of injury, (iii) to perform surgery, (iv) to relieve symptoms of illness or injury (excluding palliative care), (v) to reduce severity of an illness or injury, (vi) to protect against exacerbation and/or complication of an illness and/or injury which could threaten life or normal function, (vii) to perform diagnostic or therapeutic procedures”. In the Wikipedia entry on ‘acute care’ it reads: “In medical terms, care for acute health conditions is the opposite from chronic care, or longer-term care”.^6^ We, therefore, define acute care expenditure (ACE) very broadly as total current health care expenditure (HCE) minus long-term care expenditure (LTCE). Acute care is provided as inpatient or outpatient care by hospitals and medical and dental practices or other healthcare providers.

Long-term care, on the other hand, comprises (i) medical or nursing care, such as relieving pain and other symptoms, (ii) personal care services, i.e. help with activities of daily living, performed either by relatives or nursing staff and (iii) assistance services, which enable persons to live independently at home, e.g. shopping or performing housework (OECD et al., 2017, p. 91). The modes of provision of long-term care are (i) inpatient long-term care in hospitals or nursing homes requiring an overnight stay with medical supervision, (ii) day cases of long-term care delivered by the same providers, but without an overnight stay and (iii) outpatient or home-based long-term care, which typically involves providers of nursing services regularly visiting elderly people who are becoming more dependent (OECD et al., 2017, pp. 94–95).

In order to test whether Baumol’s cost disease affects long-term care and acute care differently we use the same strategy as Hartwig and Sturm (2014), i.e. Extreme Bounds Analysis (EBA) combined with an outlier-robust MM estimator. As EBA includes many (if not all) of the HCE drivers that have been suggested in the literature, and omitted variables are an important source of endogeneity, considering many explanatory variables mitigates the latter. However, we do not claim to properly identify causal effects. When we use the term ‘effect’ in our empirical analysis and often when we refer to the literature, it relates to conditional correlations, not a causal relationship. In other words, we are suggesting rather than testing for causal relationships.

The remainder of this paper is structured as follows. The next section discusses our dataset. Sect. "Methodology" explains the methodologies of Extreme Bounds Analysis and outlier-robust MM-estimation. Sects. "Results" and "Robustness tests" present the results—including those of robustness checks—and Sect. "Conclusion" concludes. Data issues around LTCE are discussed in Appendix 1.

## Data

The data source for most of the variables is the OECD Health Database, which also contains economic, socio-demographic, and even technological data (as long as they are health-related).^7^ Considering the dependent variables, data on total health expenditures are available for quite a long time period and many countries while data on long-term care expenditures (LTCE) tend to be relatively scarce. We exclude Ireland, Italy, New Zealand, and the United Kingdom (because of the small number of observations) and countries with a very low share (smaller than 5 percent in 2017) of LTCE in HCE (Australia, Greece, Hungary, Latvia, Portugal, and Slovakia) as their data might be of low quality and/or noisy. Our dataset thus covers the following 23 OECD countries: Austria, Belgium, Canada, the Czech Republic, Denmark, Estonia, Finland, France, Germany, Iceland, Israel, Japan, South Korea, Lithuania, Luxembourg, the Netherlands, Norway, Poland, Slovenia, Spain, Sweden, Switzerland, and the United States.

With respect to the explanatory variables, numerous possible determinants have been introduced. We draw on Hartwig and Sturm (2014), who have conducted an extensive literature review to uncover *all* macroeconomic and institutional determinants of HCE growth that have been suggested in the literature, and introduce them in an EBA framework to be explained in the next section.^8^ Most of these variables are also drawn from the OECD Health Database. Against Hartwig and Sturm (2014), we updated the sample as far as possible and included sugar intake, the importance of which as HCE driver was demonstrated by Castro (2017).^9^ Our sample period runs from 1971 to 2019.

Variable descriptions and summary statistics are given in Tables 1 and 2.

**Table 1:** Variable descriptions

Variable code	Variable label	Description	Source	Transformation
Baumol	Baumol	Compensation of employees as percentage of gross value added	OECD	Difference of log
gdp	GDP p.c	GDP per capita in US-dollars PPP	OECD	Difference of log
pop65	Population ≥ 65 years	Share of population 65 years and over	OECD/Eurostat	First difference
pop80	Population ≥ 80 years	Share of population 80 years and over	OECD/Eurostat	First difference
frp1564	Female p.r	Female participation in the labor force (% of active pop.)	OECD	First difference
ur	Unemployment rate	Unemployed as a share of the labor force (%)	OECD	First difference
ta	Health administration spending	Per capita real expenditure on health administration, governance and health system and financing administration, per capita, constant prices, OECD base year	OECD	Difference of log
accident	Road fatalities	Land traffic accidents, deaths per 100,000 population	OECD	Difference of log
alcc	Alcohol consumption	Alcohol intake, liters per capita 15 +	OECD	Difference of log
dp	Population density	Population density per square kilometer	OECD	First difference
LE65.F	Female life expectancy (65)	Life expectancy at age 65 for females	OECD	Difference of log
LE65.M	Male life expectancy (65)	Life expectancy at age 65 for males	OECD	Difference of log
mort	Mortality rate (0–69)	Mortality rate, potential years of life lost per 100 000 population, 0–69	OECD	First difference
tobc	Tobacco consumption	Tobacco consumption, grams per capita per year 15 +	OECD	Difference of log
covero	Insurance coverage	Insurance coverage % of total population covered	OECD	First difference
dl.gerd	Health R&D	Gross expenditure on R&D, compound annual growth rate	OECD	–
sugar	Sugar supply	Sugar supply in kilograms per capita per year	OECD	Difference of log
bedsi	Curative beds (per 1,000)	Curative care beds per 1000 inhabitants	OECD	Difference of log
bedsh	Curative beds (per hospital)	Curative care beds per general hospital	OECD	Difference of log
gsh	Public expenditure	Public expenditure as percentage of GDP	OECD	One year lagged first difference
puhes	Public-to-health expenditure ratio	Public expenditure as a share of total health expenditure	OECD	First difference
texmc	Inpatient expenditure	Share of inpatient expenditure in total health expenditure	OECD	First difference
hpi	Health price index	Price index for total expenditure on health	Eurostat	Difference of log
doctca	Physicians	The stock of practicing physicians per 1000 population	OECD	Difference of log
nurca	Nurses	Number of actively employed nurses per 1000 population	OECD	Difference of log
persh	Hospital employment	Total hospital employment	OECD	Difference of log
physh	Physicians per 100 beds	Physicians per 100 hospital beds	OECD	Difference of log
rat	Specialist-to-GP ratio	The ratio of specialist to general practitioners	OECD	First difference
rend	Renal dialysis	Renal dialysis rate per million inhabitants	OECD	Difference of log

**Table 2:** Summary statistics

Variable label	Observations	Mean	SD	Min	Max
Total health care expenditure*	581	0.5	4.2	− 27.8	23.0
Long-term care expenditure*	581	5.2	25.1	− 24.8	487.2
Acute care expenditure*	581	0.2	3.9	− 28.5	12.7
Baumol*	580	0.0	2.4	− 13.5	13.2
GDP p.c.*	581	1.8	2.7	− 15.4	11.7
Population ≥ 65 years**	581	0.2	0.2	− 0.4	1.0
Population ≥ 80 years**	579	0.1	0.1	− 0.4	0.7
Female p.r.**	574	0.5	0.8	− 2.6	7.7
Unemployment rate**	480	− 0.1	1.2	− 4.4	8.1
Health administration spending*	572	3.1	17.0	− 99.4	166.5
Road fatalities*	580	− 4.6	15.2	− 133.3	137.6
Alcohol consumption*	576	− 0.2	4.3	− 27.1	26.2
Population density**	480	1.0	1.6	− 4.5	8.1
Female life expectancy (65)*	575	0.7	1.4	− 5.7	8.1
Male life expectancy (65)*	575	0.9	1.4	− 6.7	8.1
Mortality rate (0–69)*	531	− 1.1	21.8	− 127.9	94.1
Tobacco consumption*	398	− 2.5	10.5	− 80.8	86.0
Insurance coverage**	520	0.1	1.7	− 5.6	36.4
Health R&D*	486	4.2	6.3	− 17.7	56.7
Sugar supply*	555	0.9	9.3	− 37.9	115.8
Curative beds (per 1,000)*	419	− 1.3	7.1	-53.2	103.2
Curative beds (per hospital)*	419	− 0.7	8.5	− 93.2	53.9
Public expenditure**	575	0.1	0.4	− 1.0	5.9
Public-to-health expenditure ratio**	581	0.1	1.9	− 7.8	35.2
Inpatient expenditure**	580	− 0.1	1.9	− 35.2	7.8
Health price index*	365	2.3	3.0	− 7.9	27.0
Physicians*	435	1.5	2.5	− 14.7	29.4
Nurses*	369	1.7	2.9	− 9.0	20.7
Hospital employment*	304	1.0	14.1	− 94.4	131.7
Physicians per 100 beds**	329	− 0.3	12.4	− 171.7	18.2
Specialist-to-GP ratio**	406	0.0	0.5	− 2.2	4.8
Renal dialysis*	410	2.5	9.7	− 116.8	36.4

*indicates difference of log, which is represented with the prefix “dl_” throughout the paper. ** indicates first difference, which is represented with the prefix “d_” throughout the paper

## Methodology

### Baseline results

To produce baseline results we regress the growth rate of HCE/ACE/LTCE in real terms on the growth rate of real GDP per capita and on the Baumol variable (i.e. the growth rate of the wage share). We include real GDP because of the longstanding insight originating from Newhouse (1977) that GDP (or income) drives HCE. Research into the determinants of HCE growth since Newhouse’s pioneering study has for a long time failed to disclose other robust explanatory variables beyond national income growth (see Roberts, 1999). Therefore, we include only GDP growth and the Baumol variable in our baseline model as well as in the M-vector of the Extreme Bounds Analysis to be discussed below.

The models are estimated with OLS using country-clustered robust standard errors as well as with an outlier-robust MM estimator. The following specifications are estimated: the first model includes only a constant, the second one additionally fixed country effects (FE country) and the third model fixed year effects (FE time). The fourth specification includes both fixed country and year effects (FE both). Furthermore, a fifth OLS specification incorporating country-specific trends (CST) is included.^10^

### Extreme bounds analysis

To examine the sensitivity of the individual variables on per-capita HCE growth, we apply (variants of) EBA, as suggested by Leamer (1985) and Levine and Renelt (1992).^11^ This approach, which has been widely used in the economic growth literature, has become a popular tool for economists who want to test the robustness of the results of their empirical work. In addition, the EBA provides an opportunity to test whether a particular determinant is robustly related to the dependent variable.

The central difficulty in this research—which also applies to the research topic of the present paper—is that several different models may all seem reasonable given the data but yield different conclusions about the parameters of interest. As argued by Temple (2000), it is rare in empirical research that we can say with certainty that one model dominates all other possibilities in all dimensions. In these circumstances, it makes sense to provide information about how sensitive the findings are to alternative modelling choices. EBA provides a relatively simple means of doing exactly this. It involves systematically testing all possible combinations of variables in a regression model. Specifically, the EBA involves running a large number of regressions, each with a different combination of variables to see how sensitive the estimated coefficients are to changes in the specification. For each regression, the coefficient of interest and the associated t-statistic are recorded. Finally, the distribution of these coefficients and t-statistics across all the regressions is examined to determine whether the coefficient of interest is robust to changes in the specification. Equations of the following general form are estimated:

1 $$Y = \, \alpha M + \, \beta F + \, \gamma Z + u,$$

where *Y* is the dependent variable; *M* is a vector of ‘standard’ explanatory variables that will be included in each regression model; *F* is the variable of interest; *Z* is a vector of up to three possible additional explanatory variables, which the literature suggests may be related to the dependent variable; and *u* is an error term. The extreme bounds test for variable *F* states that if the lower extreme bound for *β*—the lowest value for *β* minus two standard deviations—is negative, and the upper extreme bound for *β*—the highest value for *β* plus two standard deviations—is positive, the variable *F* is not robustly related to *Y*.

A main limitation of the method of EBA is that it cannot decently cope with strong multicollinearity. Two highly correlated variables often turn individually insignificant when entered jointly and should therefore ideally not both enter the EBA. Given the large number of explanatory variables included across the various model specifications in our panel dataset, there is a clear risk of multicollinearity. Before each iteration, we therefore compute the Variance Inflation Factor (VIF), which is an index that measures how much the variance of a regression coefficient is increased because of collinearity, for all included regressors. Following Hlavac (2016), we impose a maximum acceptable VIF of 7: if any variable in the specification exceeds this threshold, we exclude that particular specification from our EBA sample.

Still, the approach has been criticized in the literature.^12^ In response to the criticisms, researchers have developed several modifications of the EBA to address some of these issues. Sala-i-Martin (1997) argues that the test applied poses too rigid a threshold in most cases. Assuming that the distribution of *β* has at least some positive and some negative support, the estimated coefficient changes signs if enough different specifications are considered. We therefore report not just the lowest and highest coefficient estimates, but also the percentage of the regressions in which the coefficient of the variable *F* is significantly different from zero at the 10 percent level. Moreover, instead of analysing just the extreme bounds of the estimates of the coefficient of a particular variable, we follow Sala-i-Martin’s (1997) suggestion to analyse the entire distribution. Following this suggestion, we not only report the unweighted average parameter estimate of *β*, but also the unweighted cumulative distribution function (CDF(0)), that is, the fraction of the cumulative distribution function lying on one side of zero.

By including up to three additional variables from the ‘Z vector’, we estimate 2,952 OLS specifications for each ‘F vector’ variable; the two ‘M vector’ variables—the (growth rate of the) Baumol variable and real GDP growth—are of course always included. In this way, a total of 79,704 regressions were run for the 27 variables. Since the MM estimation is very time-consuming, we restrict the maximum number of additional ‘Z vector’ variables to two. In this case, each ‘F vector’ variable requires 352 estimations, resulting in a total of 9,504 regressions for the MM-EBA analysis.

Since our panel setup is unbalanced and contains a substantial number of missing observations, we chose not to use extensions of the EBA approach, like Bayesian Averaging of Classical Estimates (BACE), as introduced by Sala-i-Martin et al. (2004), or Bayesian Model Averaging (BMA).^13^

### Outlier-robust MM-estimation

Whereas EBA or any of its alternatives can deal with model uncertainty, i.e. whether results are robust to the selection of covariates, it does not take care of the inclusion of so-called outlying, or unusual, observations. Outlier-robust estimators can be thought of as trying to seek out the most coherent part of the data, the part best approximated by the model being estimated.

In the usual presentation of outliers, it is stressed that one or more observations may be measured with a substantial degree of error. As Swartz & Welsch (1986, p. 171) put it: “OLS and many other commonly used maximum likelihood techniques have an unbounded influence function; any small subset of the data can have an arbitrarily large influence on their coefficient estimates. In a world of fat-tailed or asymmetric error distributions, data errors and imperfectly specified models, it is just those data in which we have the least faith that often exert the most influence on the OLS estimates.”

Following Barnett & Lewis (1994, p. 316), we define an outlier as an observation ‘lying outside’ the typical relationship between the dependent and explanatory variables revealed by the remaining data. Over the last few years, several robust-to-outliers methods have been proposed in the statistical literature. High break-down point estimators, like Least Median of Squares, Least Trimmed Squares, or so-called S-estimators are able to resist a contamination of up to 50 percent of outliers. However, these estimators are known for their low efficiency at a Gaussian error distribution. To cope with this, Yohai (1987) introduced MM-estimators that combine a high-breakdown point and a high efficiency (Verardi & Croux, 2008).

Instead of squaring the residuals in the minimization process as done with OLS, within the class of S- and MM-estimators each residual undergoes a transformation dampening the influence of large residuals. For the MM-estimator, this normalizing scale is robustly determined in a first step using a so-called S-estimator that has excellent robustness properties. We use the algorithm as implemented by Koller & Stahel (2011), using a bi-square redescending score function that provides a 50% breakdown point and 95% asymptotic efficiency for normal errors.^14^ It starts by randomly picking *N* subsets of *k* observations, where *k* is the number of regression parameters to estimate.^15^ For each subset, the equation that fits all points perfectly is obtained yielding a trial solution of an outlier robust S-estimator with a Tukey biweight parameter set to 1.548.^16^ On the basis of the residuals, a scale estimate is obtained for each subset. An approximation for the scale estimate to be used in the final M-estimation is then derived from the subset that leads to the smallest scale. As far as inference is concerned, standard errors robust to heteroskedasticity are computed according to the formulas available in the literature (see Croux et al., 2008).

### Further robustness tests

To further test the robustness of our results to potential outliers or influential units in our panel data, we perform a jackknife test on our baseline models (shown in Tables 3, 4 and 5) by systematically excluding one country from our sample of 23 countries in each iteration. This procedure allows us to create 23 subsets of data, each with a different country omitted, resulting in a series of partial estimates. For each of these 23 subsets we estimate and store the coefficients on the Baumol and GDP variables and their associated *p* values using outlier-robust MM-estimation with country and time fixed effects and clustered standard errors. In this way we can assess the impact of specific countries on our results and provide insights into both the overall robustness of our statistical inferences and the sensitivity of our analysis to the exclusion of cross-section units.

**Table 3:** HCE—Baseline sample

	OLS					MM			
	Constant	FE country	FE time	FE both	CST	Constant	FE country	FE time	FE both
(Intercept)	* − *0*.*265 (0*.*207)					− 0*.*131 (0*.*139)			
dl.gdp	0*.*411^*∗∗∗*^ (0*.*067)	0*.*381^*∗∗∗*^ (0*.*075)	0*.*599^*∗∗∗*^ (0*.*086)	0*.*635^*∗∗∗*^ (0*.*115)	0*.*638^*∗∗∗*^ (0*.*087)	0*.*363^*∗∗∗*^ (0*.*045)	0*.*340^*∗∗∗*^ (0*.*054)	0*.*500^*∗∗∗*^ (0*.*061)	0*.*503^*∗∗∗*^ (0*.*104)
dl.Baumol	0*.*654^*∗∗∗*^ (0*.*079)	0*.*670^*∗∗∗*^ (0*.*077)	0*.*649^*∗∗∗*^ (0*.*091)	0*.*687^*∗∗∗*^ (0*.*072)	0*.*688^*∗∗∗*^ (0*.*073)	0*.*796^*∗∗∗*^ (0*.*051)	0*.*769^*∗∗∗*^ (0*.*063)	0*.*724^*∗∗∗*^ (0*.*058)	0*.*760^*∗∗∗*^ (0*.*085)
Num. obs	580	580	580	580	580	580	580	580	580
R^2^	0*.*172	0*.*182	0*.*362	0*.*389	0*.*528	0*.*157	0*.*285	0*.*340	0*.*440
Adj. R^2^	0*.*169	0*.*180	0*.*302	0*.*332	0*.*436				
*p* value versus none		0	0	0					
*p* value CST					0				

This table displays the estimated coefficients, their corresponding standard deviations, and *p* values for the growth rate of GDP and Baumol using HCE growth as the dependent variable. The models are estimated using OLS with country-clustered robust standard errors and an outlier-robust MM estimator. We estimated four specifications: a constant-only model; a model incorporating fixed country effects (FE country); one with fixed year effects (FE time); and a model with both fixed country and year effects included (FE both). We further estimated a fifth OLS specification that incorporated country-specific trends (CST). Robust standard errors in parentheses: *** *p* < 0.01, ** *p* < 0.05, * *p* < 0.1

**Table 4:** ACE—Baseline sample

	OLS					MM			
	Constant	FE country	FE time	FE both	CST	Constant	FE country	FE time	FE both
(Intercept)	− 0*.*57^*∗∗∗*^ (0.20)					* − *0*.*21 (0.14)			
dl.gdp	0*.*39^*∗∗∗*^ (0*.*06)	0*.*36^*∗∗∗*^ (0*.*07)	0*.*59^*∗∗∗*^ (0*.*08)	0*.*63^*∗∗∗*^ (0*.*10)	0*.*63^*∗∗∗*^ (0*.*09)	0*.*36^*∗∗∗*^ (0*.*04)	0*.*36^*∗∗∗*^ (0*.*05)	0*.*48^*∗∗∗*^ (0*.*07)	0*.*49^*∗∗∗*^ (0*.*08)
dl.Baumol	0*.*61^*∗∗∗*^ (0*.*08)	0*.*63^*∗∗∗*^ (0*.*09)	0*.*59^*∗∗∗*^ (0*.*09)	0*.*63^*∗∗∗*^ (0*.*08)	0*.*64^*∗∗∗*^ (0*.*07)	0*.*77^*∗∗∗*^ (0*.*05)	0*.*75^*∗∗∗*^ (0*.*06)	0*.*67^*∗∗∗*^ (0*.*06)	0*.*75^*∗∗∗*^ (0*.*06)
Num. obs	580	580	580	580	580	580	580	580	580
_R_2	0*.*17	0*.*18	0*.*40	0*.*43	0*.*57	0*.*15	0*.*27	0*.*32	0*.*42
Adj. R^2^	0*.*17	0*.*18	0*.*34	0*.*38	0*.*48				
*p* value versus none		0	0	0					
*p* value CST					0				

This table displays the estimated coefficients, their corresponding standard deviations, and *p* values for the growth rate of GDP and Baumol using ACE growth as the dependent variable. The models are estimated using OLS with country-clustered robust standard errors and an outlier-robust MM estimator. We estimated four specifications: a constant-only model; a model incorporating fixed country effects (FE country); one with fixed year effects (FE time); and a model with both fixed country and year effects included (FE both). We further estimated a fifth OLS specification that incorporated country-specific trends (CST). Robust standard errors in parentheses: *** *p* < 0.01, ** *p* < 0.05, * *p* < 0.1

**Table 5:** LTCE—Baseline sample

	OLS					MM			
	Constant	FE country	FE time	FE both	CST	Constant	FE country	FE time	FE both
(Intercept)	2*.*62^*∗∗∗*^ (0*.*78)					0*.*95^*∗∗∗*^ (0*.*24)			
dl.gdp	1*.*42^*∗∗*^ (0*.*60)	0*.*85^*∗∗*^ (0*.*37)	2*.*33^*∗∗*^ (1.13)	1*.*33^*∗*^ (0*.*66)	0*.*67^*∗∗*^ (0.32)	0*.*20^*∗∗*^ (0*.*09)	0*.*25^*∗∗∗*^ (0*.*09)	0*.*22 (0*.*16)	0*.*25 (0*.*18)
dl.Baumol	0*.*92^*∗∗∗*^ (0*.*22)	0*.*69^*∗∗∗*^ (0*.*22)	0*.*85^*∗∗*^ (0*.*38)	0*.*68^*∗∗*^ (0*.*28)	0*.*66^*∗∗*^ (0*.*29)	0*.*71^*∗∗∗*^ (0*.*08)	0*.*77^*∗∗∗*^ (0*.*09)	0*.*75^*∗∗∗*^ (0*.*13)	0*.*85^*∗∗∗*^ (0*.*12)
Num. obs	580	580	580	580	580	580	580	580	580
R^2^	0*.*03	0*.*01	0*.*09	0*.*07	0*.*16	0*.*06	0*.*24	0*.*17	0*.*31
Adj. R^2^	0*.*02	0*.*01	0.00	− 0*.*02	0*.*04				
*p* value versus none		0	0.85	0					
*p* value CST					0				

This table displays the estimated coefficients, their corresponding standard deviations, and *p* values for the growth rate of GDP and Baumol using LTCE growth as the dependent variable. The models are estimated using OLS with country-clustered robust standard errors and an outlier-robust MM estimator. We estimated four specifications: a constant-only model; a model incorporating fixed country effects (FE country); one with fixed year effects (FE time); and a model with both fixed country and year effects included (FE both). We further estimated a fifth OLS specification that incorporated country-specific trends (CST). Robust standard errors in parentheses: *** *p* < 0.01, ** *p* < 0.05, * *p* < 0.1

In addition, we re-estimate EBAs with all independent variables lagged by one year to address possible endogeneity from reverse causality.^17^ Lagged control variables should attenuate potential reverse causality. Although we do not think that health care expenditure has a significant impact on the Baumol variable, which measures the (growth of) the wage share in GDP, we carry out this procedure as a precautionary measure. Even though this robustness test cannot properly identify causal effects, we believe that it adds evidential weight to the argument that they might exist.

## Results

### Baseline results

The results for HCE as dependent variable (Table 3) confirm the finding of Hartwig and Sturm (2014) that the coefficient on the Baumol variable is greater than the coefficient on GDP growth. Yet there are considerable differences between the OLS and MM results. With outlier-robust methods, the GDP effect tends to be smaller and the Baumol effect greater than with OLS. It is striking that in all specifications, both the GDP and Baumol coefficients are significant at the 1% level. The coefficients on the Baumol variable obtained using OLS range between 0.65 and 0.69, while MM provides a higher range of 0.72 to 0.80. The estimated GDP coefficients are somewhat more volatile and range between 0.38–0.64 and 0.34–0.50 respectively.

The regressions with ACE as dependent variable (Table 4) mirror the HCE results in terms of the estimated coefficients and their significance: the Baumol coefficients are usually greater than the GDP coefficients, with all coefficients of both variables being significant at the 1% level. Again, the range of coefficients in the case of OLS is smaller compared to the MM results, with the GDP coefficients again exhibiting greater volatility.

The picture changes somewhat with LTCE as dependent variable (Table 5). The estimated GDP coefficients, which exhibit relatively high volatility—they range between 0.67 and 2.33 in the OLS estimations –, are insignificant in two out of four MM specifications. The Baumol coefficients are less volatile (OLS: 0.68–0.92; MM: 0.71–0.85) and are always significant at least at the 5% level. In order to check whether GDP and the Baumol variable affect LTCE and ACE differently, we conduct a Wald test using seemingly unrelated estimation to combine estimates from multiple models (see Tables 6 and 7). We find little evidence that the impact of either GDP or the Baumol variable on ACE is much different than their impact on LTCE.

**Table 6:** Tests—Baumol coefficient LTCE versus ACE

	OLS					MM			
	Constant	FE country	FE time	FE both	CST	Constant	FE country	FE time	FE both
Test-statistic	1.923	0.032	0.517	0.012	0.009	− 0.567	0.236	0.564	0.833
*P* value	0.166	0.859	0.472	0.914	0.924	0.285	0.593	0.713	0.797

This table presents the test statistics and corresponding *p* values of a Wald test which assesses whether the estimated coefficient of the Baumol variable is equal in the ACE and LTCE estimates

**Table 7:** Tests—GDP coefficient LTCE versus ACE

	OLS					MM			
	Constant	FE country	FE time	FE both	CST	Constant	FE country	FE time	FE both
Test-statistic	2.951	1.577	2.497	0.952	0.005	− 1.681	− 1.145	− 1.440	− 1.269
*P* value	0.086	0.209	0.114	0.329	0.945	0.047	0.126	0.075	0.103

This table presents the test statistics and corresponding *p* values of a Wald test which assesses whether the estimated coefficient of GDP is equal in the ACE and LTCE estimates

### Results from extreme bounds analysis (EBA)

Table 8 shows the results—which are ordered based on their CDF(0) in the OLS regressions—for the EBA with HCE as dependent variable. Overall, the OLS and MM estimations confirm the results of the baseline calculations. The Baumol and GDP variables tend to be the most robust correlates of health care expenditure growth: in both OLS and MM, their CDF(0) and share of significant coefficients equals 100, which means that for all regressions the coefficient signs are on one side of zero and significant at least at the 5% level. When estimated with OLS (MM), the average coefficient values of the Baumol and GDP variables are 0.69 (0.72) and 0.61 (0.59), respectively, which is in line with the baseline results.

**Table 8:** EBA results for HCE (with year and country FE)

	OLS						MM					
Variables	Mean *β*	Min *β*	Max *β*	Ø se	%Sign	CDF(0)	Mean *β*	Min *β*	Max *β*	Ø se	%Sign	CDF(0)
dl.Baumol	0.69	0.53	0.84	0.07	**100.0**	**100.0**	0.72	0.60	0.85	0.08	**100.0**	**100.0**
dl.gdp	0.61	0.34	1.18	0.10	**100.0**	**100.0**	0.59	0.38	1.26	0.10	**100.0**	**100.0**
d.ur	0.40	0.04	1.16	0.21	37.5	**100.0**	0.48	0.28	0.99	0.19	76.6	**100.0**
dl.hpi	− 0.15	− 0.32	0.02	0.06	63.1	**99.1**	0.00	− 0.15	0.12	0.07	0.6	57.0
dl.tobc	0.01	0.00	0.02	0.01	0.0	**98.1**	0.01	0.00	0.02	0.01	0.7	**98.7**
d.gsh_l1	0.60	− 0.26	2.78	0.46	12.7	**96.0**	0.61	0.14	2.18	0.32	25.5	**100.0**
d.physh	0.02	− 0.04	0.12	0.02	33.8	**93.0**	0.03	− 0.06	0.10	0.02	30.2	**93.4**
dl.sugar	0.02	− 0.02	0.04	0.02	0.0	**91.1**	0.00	− 0.01	0.02	0.01	0.0	74.4
dl.bedsh	0.01	− 0.16	0.06	0.02	1.6	86.4	0.00	− 0.04	0.03	0.02	0.0	79.2
dl.ta	0.01	− 0.02	0.08	0.01	22.5	85.7	0.01	− 0.01	0.06	0.01	13.2	**95.6**
d.puhes	0.28	-2.53	16.75	0.24	3.7	83.2	0.07	− 2.31	7.69	0.08	3.0	56.8
d.rat	− 0.14	− 0.81	0.52	0.37	0.0	82.8	− 0.27	− 0.59	0.18	0.35	3.4	**98.6**
dl.persh	0.01	− 0.04	0.10	0.02	13.6	82.2	0.02	− 0.04	0.07	0.02	17.9	85.5
dl.accident	0.00	− 0.03	0.01	0.01	2.1	81.9	− 0.01	− 0.02	0.00	0.01	0.8	**100.0**
d.texmc	0.20	− 2.54	16.71	0.24	3.0	81.8	0.06	− 2.33	7.69	0.08	3.0	53.8
d.pop80	1.03	− 3.15	6.66	2.16	0.5	80.9	0.09	− 3.64	2.31	1.52	0.0	53.2
dl.LE65.M	− 0.10	− 0.37	0.40	0.13	5.3	80.6	− 0.03	− 0.19	0.19	0.12	0.0	60.2
dl.gerd	− 0.01	− 0.07	0.07	0.03	0.3	79.1	− 0.01	− 0.04	0.06	0.03	0.0	83.2
dl.nurca	0.02	− 0.08	0.14	0.07	0.0	78.6	0.02	− 0.03	0.15	0.04	1.6	83.3
d.frp1564	0.14	− 0.29	0.68	0.24	0.0	78.4	− 0.08	− 0.30	0.11	0.21	0.0	66.4
d.mort	0.01	− 0.02	0.04	0.01	2.6	78.1	0.00	0.00	0.02	0.01	0.0	**96.4**
dl.doctca	− 0.05	− 0.29	0.22	0.09	11.0	74.6	− 0.08	− 0.23	0.20	0.09	1.1	**97.8**
dl.bedsi	0.01	− 0.11	0.11	0.03	0.7	73.5	0.03	− 0.03	0.07	0.03	8.6	**97.1**
d.dp	0.07	− 0.36	0.61	0.26	0.0	69.7	0.01	− 0.24	0.48	0.17	0.5	52.2
dl.LE65.F	− 0.08	− 0.55	0.47	0.16	6.5	67.0	− 0.02	− 0.37	0.27	0.13	0.8	62.0
dl.alcc	0.02	− 0.04	0.28	0.04	3.5	65.2	0.01	− 0.01	0.08	0.03	0.0	71.3
dl.rend	− 0.01	− 0.05	0.04	0.02	5.4	62.3	0.00	− 0.03	0.05	0.02	0.0	60.7
d.covero	− 0.01	− 0.49	2.72	0.23	0.0	62.0	0.14	− 0.19	2.25	0.17	7.3	66.0
d.pop65	0.18	− 2.87	2.59	1.08	0.1	57.6	0.04	− 0.74	0.93	0.75	0.0	51.4

This table shows the results of OLS-EBA and MM-EBA estimations using HCE growth as the dependent variable. For the explanatory variables, the average estimated coefficient (mean *β*), minimum and maximum of the coefficients (min *β* and max *β*), their average standard deviation (Ø se), the proportion of significant coefficients at the 5% level (%Sign.) and the cumulative distribution function CDF(0) of the estimated coefficients are reported. CDF(0) values > 90% and %Sign values > 90% are highlighted in bold

Sala-i-Martin (1997) suggests that explanatory variables count as robust when their CDF(0) > 90%. According to this criterion, six additional variables—apart from Baumol and GDP growth—are robust in the OLS-EBA and ten in the MM-EBA. Although most of them are not statistically significant very often and the signs on the coefficients are sometimes dubious,^18^ further variables also demonstrate somewhat robust and significant relationships with health care expenditure growth in both OLS and MM analyses. The first difference of the unemployment rate (d.ur) is positively associated with health care expenditure growth, with an average coefficient indicating that a 1 percentage point increase in the unemployment rate leads to an increase in health care expenditure growth between 0.4 (OLS) and 0.5 (MM) percentage points. Similarly, one-year lagged public expenditure as a percentage of GDP (d.gsh_l1) shows a positive and significant relationship, suggesting that a 1 percentage point increase in this share raises HCE growth by about 0.6 percentage points. Another notable correlate is the number of physicians per 100 hospital beds (d.physh), which is also positively associated with health care expenditure growth according to both estimation methods. The effect of an increase of one physician per 100 hospital beds on HCE growth is small, however. The overall picture emerging from Table 8 confirms the findings of Hartwig and Sturm (2014).

In this paper, we go beyond Hartwig and Sturm (2014) in testing whether Baumol’s disease affects acute care and long-term care differently. The first thing to note from Table 9 is that the results for ACE are similar to those reported for overall HCE in Table 8. In the OLS- and MM-EBAs, the growth rates of GDP and the Baumol variable emerge as robust and always significant explanatory variables for ACE growth. The change in the unemployment rate (d.ur) and the growth in per capita real expenditure on health administration (dl.ta) emerge as robust cost drivers from the MM-EBA that are statistically significant in more than the half of the regressions they enter. The average coefficients suggest that a one percentage point increase in the unemployment rate is associated with a 0.4–0.5 percentage point rise in ACE growth. The MM estimation furthermore highlights that an increase in per capita real expenditure on health administration (dl.ta) leads to a small, but often significant rise in overall ACE.

**Table 9:** EBA results for ACE (with year and country FE)

	OLS						MM					
Variables	Mean *β*	Min *β*	Max *β*	Ø se	%Sign	CDF(0)	Mean *β*	Min *β*	Max *β*	Ø se	%Sign	CDF(0)
dl.Baumol	0.64	0.50	0.84	0.07	**100.0**	**100.0**	0.69	0.55	0.85	0.08	**100.0**	**100.0**
dl.gdp	0.62	0.34	1.17	0.09	**100.0**	**100.0**	0.57	0.34	1.20	0.10	**100.0**	**100.0**
d.ur	0.39	0.05	1.27	0.20	48.5	**100.0**	0.49	0.30	0.98	0.22	67.4	**100.0**
dl.tobc	0.01	0.00	0.03	0.01	0.0	**100.0**	0.01	0.00	0.02	0.01	1.4	**100.0**
dl.hpi	− 0.15	− 0.31	0.07	0.06	69.5	**97.1**	− 0.10	− 0.29	0.14	0.12	9.8	**93.0**
dl.accident	− 0.01	− 0.04	0.00	0.01	11.3	**97.0**	− 0.01	− 0.04	− 0.01	0.01	28.2	**100.0**
d.gsh_l1	0.64	− 0.30	2.87	0.43	18.8	**96.7**	0.42	0.08	1.73	0.27	8.7	**100.0**
dl.persh	0.02	− 0.04	0.08	0.01	10.5	**90.5**	0.01	− 0.02	0.07	0.02	12.2	82.7
dl.sugar	0.01	− 0.02	0.05	0.02	0.0	85.7	0.00	− 0.03	0.01	0.01	0.0	53.7
dl.gerd	− 0.02	− 0.10	0.06	0.03	3.8	82.8	− 0.01	− 0.05	0.04	0.03	1.6	81.3
dl.alcc	0.02	− 0.05	0.21	0.03	6.8	82.0	0.01	− 0.05	0.08	0.03	0.0	70.5
d.physh	0.01	− 0.07	0.10	0.02	4.0	81.5	0.02	− 0.10	0.10	0.02	15.0	**90.0**
dl.nurca	0.02	− 0.08	0.10	0.07	0.0	77.3	0.02	− 0.03	0.13	0.05	0.0	68.0
dl.ta	0.01	− 0.02	0.12	0.01	14.1	74.1	0.02	− 0.01	0.15	0.01	52.3	**99.2**
d.rat	− 0.09	− 0.79	0.83	0.35	0.1	72.3	− 0.33	− 0.77	0.23	0.41	2.8	**98.6**
dl.LE65.M	− 0.06	− 0.36	0.34	0.12	0.3	69.0	− 0.03	− 0.19	0.15	0.13	0.0	74.0
dl.bedsi	0.01	− 0.07	0.15	0.03	0.1	69.0	0.01	− 0.04	0.05	0.02	0.0	**91.5**
dl.bedsh	0.00	− 0.14	0.06	0.02	1.0	68.7	0.00	− 0.05	0.02	0.01	0.5	70.5
d.dp	0.04	− 0.30	0.59	0.23	0.0	62.4	− 0.04	− 0.23	0.33	0.20	0.0	71.3
d.frp1564	− 0.05	− 0.43	0.42	0.22	0.0	62.3	− 0.10	− 0.30	0.19	0.23	0.0	84.7
dl.LE65.F	0.00	− 0.45	0.45	0.15	0.4	56.9	0.01	− 0.29	0.35	0.13	0.0	59.5
d.covero	0.01	− 0.46	3.16	0.21	0.0	56.6	0.09	− 0.27	1.40	0.20	0.0	55.2
d.texmc	0.15	− 4.06	18.49	0.23	2.3	55.1	0.33	− 1.87	7.74	0.15	5.6	62.6
d.mort	0.00	− 0.02	0.03	0.01	0.0	54.4	0.00	− 0.01	0.02	0.01	0.0	88.3
d.pop65	− 0.11	− 2.23	2.37	1.00	0.0	53.7	0.08	− 0.61	1.40	0.81	0.0	60.2
dl.doctca	− 0.02	− 0.29	0.30	0.08	7.4	53.7	− 0.05	− 0.24	0.22	0.08	2.3	86.4
dl.rend	0.00	− 0.04	0.06	0.02	0.1	53.3	0.00	− 0.02	0.03	0.02	0.0	67.4
d.puhes	0.17	− 4.08	18.52	0.23	2.4	52.3	0.33	− 1.86	7.73	0.14	5.4	59.5
d.pop80	− 0.05	− 4.71	3.79	2.00	0.0	50.7	− 0.69	− 4.47	1.29	1.41	0.0	82.5

This table shows the results of OLS-EBA and MM-EBA estimations using ACE growth as the dependent variable. For the explanatory variables, the average estimated coefficient (mean *β*), minimum and maximum of the coefficients (min *β* and max *β*), their average standard deviation (Ø se), the proportion of significant coefficients at the 5% level (%Sign.) and the cumulative distribution function CDF(0) of the estimated coefficients are reported. CDF(0) values > 90% and %Sign values > 90% are highlighted in bold

Skepticism with regard to the relevance of Baumol’s cost disease in acute care should be soothed by these results. The rise of hi-tech medicine in acute care has not been able to cure Baumol’s disease, and the OECD’s decision to allow for “the impact of the Baumol effect on health care as a whole (instead of only for long-term care)” (Lorenzoni et al., 2019, p. 25) in their HCE projections is vindicated by our findings. Note, however, that the coefficient on the Baumol variable is a bit smaller in Table 9 than in Table 8 both for the OLS-EBA and the MM-EBA. This suggests that acute care expenditures are somewhat less prone to the Baumol effect than overall HCE. Two-sample t-tests for the estimated Baumol coefficients with OLS and MM are performed to test the statistical significance of this finding.^19^ In both cases, the difference in the Baumol coefficients between ACE and HCE is significant at the 1% level.

Inspecting the results for long-term care expenditure in Table 10 reveals notable differences. We confine ourselves to commenting the MM-EBA results, which appear to be more plausible (see below). The Baumol variable remains robust and is statistically significant in 99.9% of the regressions. It is noticeable that the coefficient on this variable is on average higher than in Tables 8 and 9, pointing—in line with prior expectations—to an especially high impact of Baumol’s cost disease in long-term care. We use a two-sample t-test to assess this finding, which shows that the difference in the Baumol coefficients between ACE and LTCE in both OLS and MM estimations is significant at the 1% level.^20^ This suggests that the Baumol coefficient is significantly higher for LTCE as dependent variable than for ACE.^21^

**Table 10:** EBA results for LTCE (with year and country FE)

	OLS						MM					
Variables	Mean *β*	Min *β*	Max *β*	Ø se	%Sign	CDF(0)	Mean *β*	Min *β*	Max *β*	Ø se	%Sign	CDF(0)
dl.tobc	− 0.04	− 0.09	0.00	0.07	0.0	**100.0**	0.00	− 0.02	0.02	0.02	0.0	87.0
dl.Baumol	0.79	− 0.29	1.59	0.51	49.5	**99.7**	0.92	0.52	1.22	0.13	**99.9**	**100.0**
dl.LE65.M	− 1.27	− 3.93	0.86	0.96	9.2	**98.0**	− 0.05	− 0.31	0.25	0.18	0.0	57.7
dl.gdp	0.88	− 0.86	3.45	0.69	7.7	**97.7**	0.41	0.10	1.27	0.21	43.5	**100.0**
d.mort	0.12	− 0.09	0.42	0.08	24.3	**95.7**	0.01	− 0.02	0.03	0.01	0.0	84.5
dl.gerd	0.38	− 0.12	0.96	0.23	32.7	**95.1**	− 0.03	− 0.09	0.20	0.05	2.4	82.0
dl.sugar	0.11	− 0.19	0.23	0.14	0.0	**95.1**	0.02	− 0.01	0.04	0.02	0.0	**92.3**
dl.ta	0.12	− 0.16	0.51	0.07	39.6	**94.1**	− 0.02	− 0.14	0.03	0.02	9.7	71.3
d.texmc	− 0.56	− 14.87	27.99	1.69	15.5	**91.0**	0.20	− 3.87	15.34	0.25	15.9	64.0
d.physh	0.08	− 0.20	0.85	0.10	0.3	**90.9**	0.02	− 0.42	0.23	0.04	27.8	50.4
d.puhes	1.07	− 13.72	30.23	1.69	14.8	**90.3**	0.21	− 3.86	15.36	0.24	18.6	54.3
dl.alcc	0.31	− 0.51	1.03	0.27	0.4	88.3	− 0.02	− 0.19	0.20	0.08	0.5	75.1
d.ur	0.98	− 1.56	3.40	1.67	0.6	87.1	0.81	0.40	1.17	0.32	87.1	**100.0**
d.pop65	9.82	− 6.69	31.89	8.01	19.1	86.9	− 1.24	− 2.83	0.77	1.29	1.9	**94.7**
dl.persh	− 0.08	− 0.36	0.24	0.09	2.1	85.1	0.01	− 0.04	0.13	0.03	7.1	69.5
d.frp1564	1.41	− 2.62	6.36	1.75	25.1	81.9	0.06	− 0.32	0.65	0.30	0.0	56.9
dl.LE65.F	− 0.43	− 2.24	5.63	1.19	3.9	79.6	− 0.21	− 0.74	0.01	0.21	7.8	**99.1**
d.dp	0.69	− 2.29	3.14	1.98	0.0	79.6	0.54	− 0.04	1.30	0.30	38.6	**99.6**
dl.nurca	− 0.29	− 1.28	0.63	0.58	0.0	76.4	0.02	− 0.05	0.25	0.06	6.1	58.2
dl.accident	0.02	− 0.09	0.11	0.07	0.0	75.5	0.02	-0.01	0.03	0.01	2.7	**99.1**
dl.doctca	− 1.38	− 7.10	0.86	0.67	56.8	69.6	− 0.18	− 0.40	0.11	0.10	64.1	**99.5**
dl.bedsi	− 0.14	− 2.19	0.56	0.28	2.4	67.7	− 0.04	− 0.26	0.59	0.06	5.4	**98.2**
dl.rend	− 0.03	− 0.21	0.10	0.10	4.4	66.9	− 0.02	− 0.15	0.04	0.03	4.2	82.1
d.pop80	− 15.49	− 85.83	28.79	16.03	5.7	65.0	6.57	1.10	11.00	3.06	68.6	**100.0**
d.gsh_l1	− 0.98	− 10.95	3.09	3.43	0.0	63.5	0.64	− 0.22	1.73	0.42	20.4	**98.1**
dl.bedsh	− 0.16	− 1.14	0.44	0.18	7.1	63.3	0.03	− 0.06	0.13	0.03	7.9	**98.7**
dl.hpi	0.39	− 1.16	1.57	0.52	17.0	53.1	− 0.14	− 0.72	0.17	0.19	4.7	**91.8**
d.covero	− 0.02	− 6.87	3.04	1.22	2.7	51.9	− 0.04	− 0.50	0.71	0.27	2.1	54.2
d.rat	− 0.28	− 4.23	5.83	3.02	0.0	50.7	− 0.60	− 1.97	0.48	0.81	8.1	89.2

This table shows the results of OLS-EBA and MM-EBA estimations using LTCE growth as the dependent variable. For the explanatory variables, the average estimated coefficient (mean *β*), minimum and maximum of the coefficients (min *β* and max *β*), their average standard deviation (Ø se), the proportion of significant coefficients at the 5% level (%Sign.) and the cumulative distribution function CDF(0) of the estimated coefficients are reported. CDF(0) values > 90% and %Sign values > 90% are highlighted in bold

The importance of income (GDP) seems to be lower in explaining long-term care expenditure compared with acute care or overall health expenditure. GDP growth remains robust, but the coefficient is lower than in Tables 8 and 9, and the variable is statistically significant in less than 50% of the regressions in the MM-EBA (and less than 10% in the OLS-EBA). Other robust variables ‘outperform’ GDP growth in terms of higher proportions of significant coefficients, for instance, the (change in the) unemployment rate (d.ur),^22^ the change in the share of the population 80 years and over (d.pop80) and the growth rate of the stock of practicing physicians per 1000 population (dl.doctca).^23^ One reason why we think the MM-EBA results are more plausible than the OLS-EBA results for LTCE is that the coefficient on d.pop80 is negative on average in the OLS estimations.

## Robustness tests

**Fig. 1:**
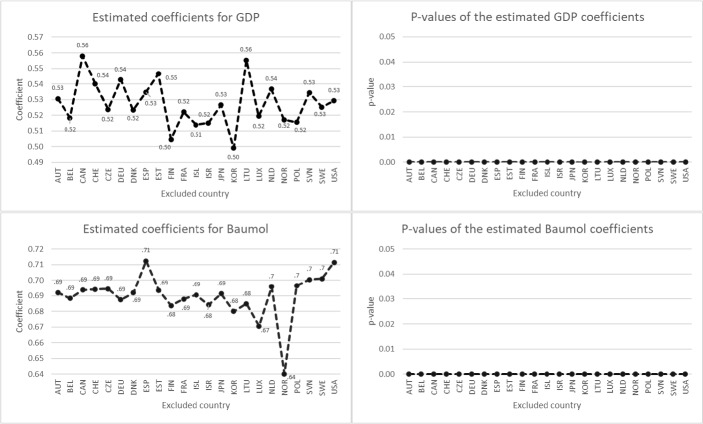
Jackknife results for HCE *Note*: The dependent variable is HCE growth. The figure shows the estimated coefficients and their corresponding *p* values for GDP growth and Baumol in each iteration, omitting one of the 23 countries in each step. The coefficients were estimated using outlier-robust MM-estimation with country and time fixed effects, using clustered standard errors to calculate the *p* values. The mean $\mu$ and standard deviation $\sigma$ of the 23 coefficients calculated by the jackknife test are ${\mu }_{GDP}=0.53$, ${\mu }_{Baumol}=0.69$, ${\sigma }_{GDP}=0.0144$ and ${\sigma }_{Baumol}=0.0138$

**Fig. 2:**
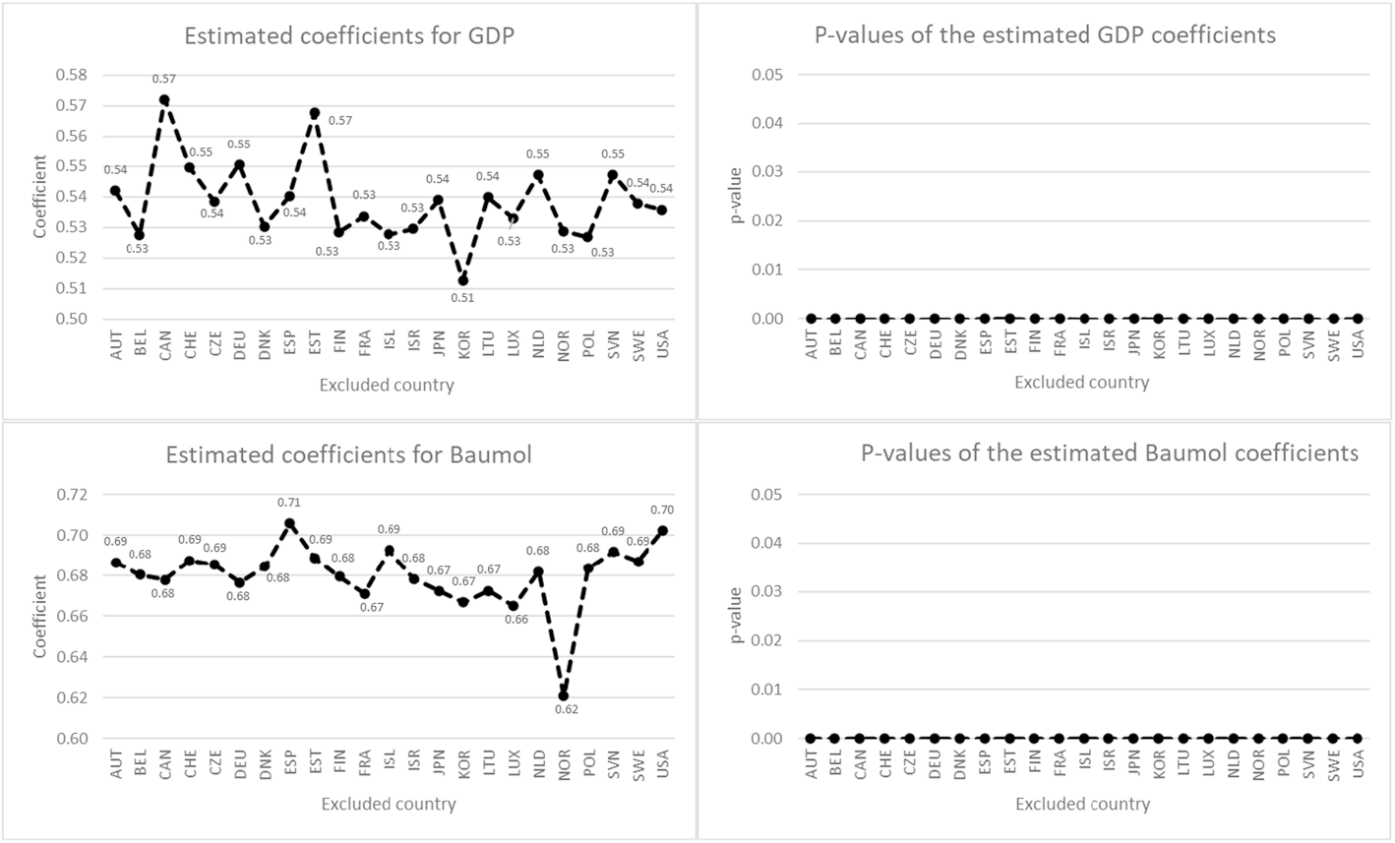
Jackknife results for ACE *Note*: The dependent variable is ACE growth. The figure shows the estimated coefficients and their corresponding *p* values for GDP growth and Baumol in each iteration, omitting one of the 23 countries in each step. The coefficients were estimated using outlier-robust MM-estimation with country and time fixed effects, using clustered standard errors to calculate the *p* values. The mean $\mu$ and standard deviation $\sigma$ of the 23 coefficients calculated by the jackknife test are ${\mu }_{GDP}=0.54$, ${\mu }_{Baumol}=0.68$, ${\sigma }_{GDP}=0.0129$ and ${\sigma }_{Baumol}=0.0159$

Figure 1 shows the results of the jackknife test for HCE. The lowest estimated coefficients for the GDP and Baumol variables are 0.50 and 0.64, respectively. These were obtained when South Korea and Norway (respectively) were excluded. The highest estimated coefficient for GDP is found by excluding Canada (0.56). For the Baumol variable, the highest coefficient (0.71) results when excluding Spain. The mean values of the 23 coefficients calculated by the jackknife test are ${\mu }_{GDP}=0.53$ and ${\mu }_{Baumol}=0.69$. With a standard deviation of 0.0144 and 0.0138 respectively, the estimated coefficients on GDP and the Baumol variable remained relatively robust throughout this experiment. It is also striking that in all iterations the estimated coefficients for both independent variables are significant at the 1% level. Results of the jackknife test are very similar when ACE instead of HCE is the dependent variable (see Fig. 2).

**Fig. 3:**
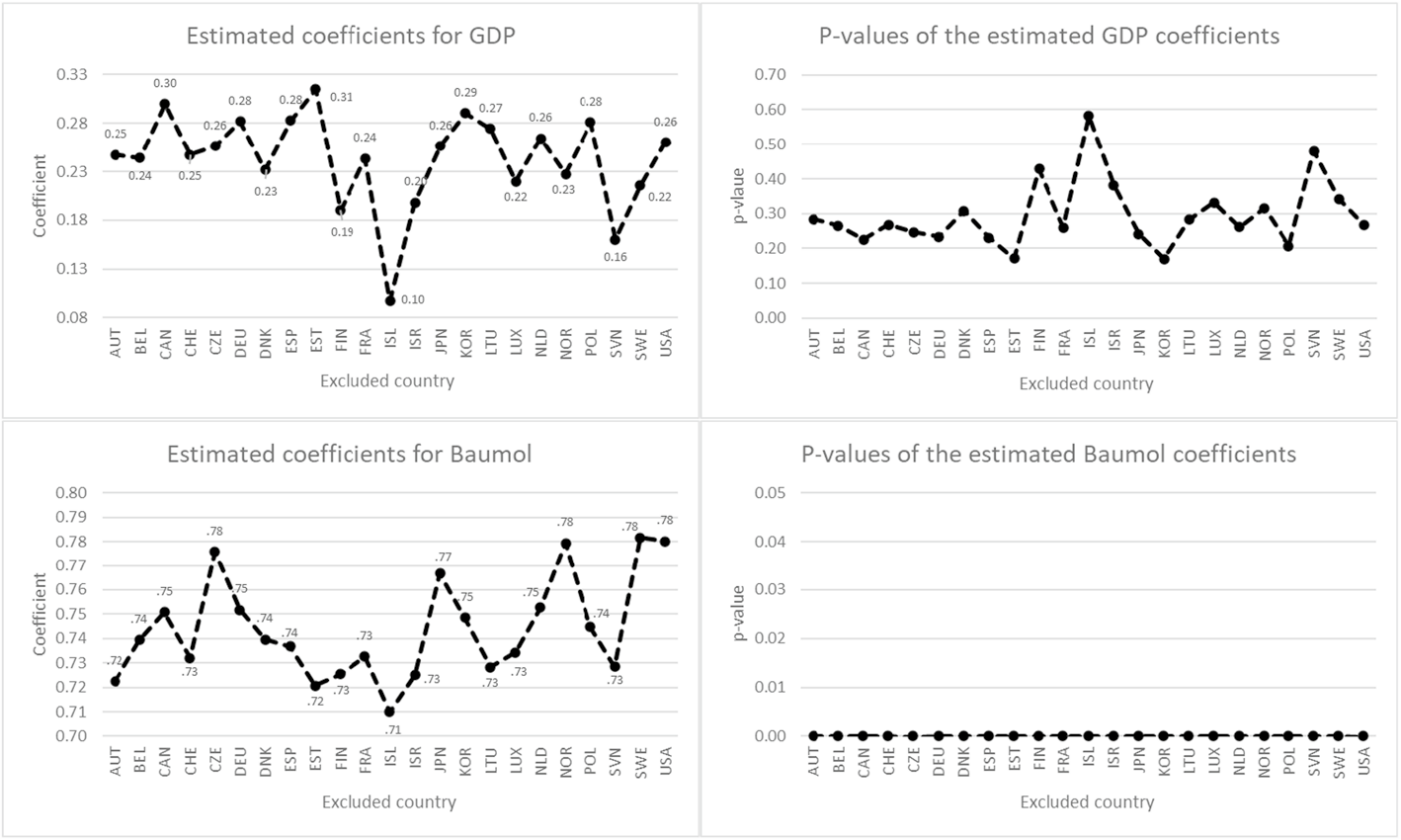
Jackknife results for LTCE *Note*: The dependent variable is LTCE growth. The figure shows the estimated coefficients and their corresponding *p* values for GDP growth and Baumol in each iteration, omitting one of the 23 countries in each step. The coefficients were estimated using outlier-robust MM-estimation with country and time fixed effects, using clustered standard errors to calculate the *p* values. The mean $\mu$ and standard deviation $\sigma$ of the 23 coefficients calculated by the jackknife test are ${\mu }_{GDP}=0.24$, ${\mu }_{Baumol}=0.74$, ${\sigma }_{GDP}=0.0475$ and ${\sigma }_{Baumol}=0.0203$

The jackknife results for LTCE as dependent variable are displayed in Fig. 3. The mean values and standard deviations of the coefficients are: ${\mu }_{GDP}=0.24$, ${\sigma }_{GDP}=0.0475, {\mu }_{Baumol}=0.74$, ${\sigma }_{Baumol}=0.0203$.^24^ Excluding Iceland results in the lowest GDP coefficient (0.10), while excluding Estonia produces the highest one (0.31). The lowest Baumol coefficient (0.71) is obtained by excluding Iceland, while the highest one (0.78) is obtained by excluding Sweden. All estimated Baumol coefficients remain statistically significant at the 1% level; GDP growth, however, does not contribute to explaining LCTE growth at conventional levels of significance. This confirms our finding from Table 5 for the outlier-robust MM-estimation with country and time fixed effects.

The results of the EBA estimations performed with lagged independent variables are shown in Tables 11, 12 and 13 in Appendix 2. When HCE or ACE are considered as dependent variables, high CDF(0) values indicate that the Baumol variable remains a robust explanatory variable, although its lagged version is statistically significant in fewer regressions than its un-lagged counterpart, and the coefficient estimates are considerably lower. Lagged GDP growth is also a quite robust explanatory variable for both HCE and ACE growth, with relatively high CDF(0) values. This is not the case for LTCE growth, however. In the LTCE EBA estimated with OLS, the lagged Baumol variable drops below the 90% CDF(0) threshold. This is due to outliers, however, as the MM estimation demonstrates. Even when lagged by one year, the Baumol variable remains a robust explanatory variable for LTCE growth.

## Conclusion

Baumol’s cost disease has been argued to be a major driver of health care expenditure growth. Skeptics doubt its impact on expenditures on acute care, however, maintaining that only labor intensive tasks in long-term care are likely to be affected by the cost disease. Our aim in this paper was to test this proposition empirically.

In our analysis, we combine Extreme Bounds Analysis (EBA) with outlier-robust MM estimation and use data for 23 OECD countries over the period 1971–2019. We find that, although the impact of Baumol’s cost disease is strongest on long-term care expenditure, expenditure on acute care is nevertheless also affected. These findings are robust to excluding single countries from the sample as well as to lagging all explanatory variables by one year.

We conclude that Baumol’s cost disease drives the whole range of health care expenditures, not just those on labor intensive care work. Our results hence give succor to the decision taken by the OECD in 2019 to revise the methodology used for the organization’s health spending projections to allow for an “impact of the Baumol effect on health care as a whole (instead of only for long-term care)” (Lorenzoni et al., 2019, p. 25).

## Appendix 1: Data issues around LTCE

As OECD et al. (2017, p. 88) note, “(t)o date, estimates of spending on services for long-term health care have been mostly limited to higher-income countries, due to the fact that in most low- and middle-income countries (LMIC) long-term health care is provided as informal care, i.e. usually consumed at home, provided by relatives and without any transaction or record involved”. Even in high-income OECD countries, “due to differing administrative, financing and provision standards, the national boundaries of long-term care may not necessarily be aligned with” the boundaries defined in the System of Health Accounts (*ibid*., p. 92). The System of Health Accounts stipulates that LTCE figures should include all expenditures, irrespective of the mode of provision or financing scheme of long-term care. However, “(d)ata for Latvia, Poland, the United States, Japan, Ireland, the Slovak Republic and Canada are only available for people receiving LTC in institutions, so the total number of recipients will be underestimated” (OECD, 2023, p. 220). Informal care, mainly provided by unpaid family members, is disregarded in LTCE figures, which impedes comparability since countries vary widely with respect to their formal LTC systems, as opposed to more informal arrangements (*ibid*., p. 228). Such data-related limitations should be borne in mind when interpreting our results.

## Appendix 2

See Tables Tables 11, 12 and 13.

**Table 11:** EBA results for HCE (with year and country FE) using one year lagged explanatory variables

	OLS						MM					
Variables	Mean *β*	Min *β*	Max *β*	Ø se	%Sign	CDF(0)	Mean *β*	Min *β*	Max *β*	Ø se	%Sign	CDF(0)
L.dl.hpi	− 0.13	− 0.25	− 0.02	0.08	48.2	**100.0**	− 0.09	-0.21	0.02	0.06	25.7	**97.9**
L.dl.sugar	0.03	0.00	0.12	0.02	21.0	**99.5**	0.01	0.00	0.06	0.02	0.0	**99.2**
L.d.pop80	− 4.83	− 13.70	4.74	2.61	54.3	**99.4**	− 2.08	− 7.13	1.16	1.79	10.1	**93.9**
L.dl.gdp	0.20	− 0.10	0.91	0.12	28.6	**99.2**	0.14	− 0.13	0.42	0.12	10.6	**96.3**
L.dl.rend	0.04	− 0.02	0.07	0.02	43.8	**99.2**	0.05	0.02	0.09	0.02	76.2	**100.0**
L.dl.Baumol	0.18	− 0.13	0.43	0.09	62.1	**98.4**	0.27	− 0.04	0.58	0.14	33.2	**99.0**
d.gsh_l1	0.58	− 0.89	2.60	0.55	10.3	**96.0**	0.62	0.08	1.34	0.31	73.3	**100.0**
L.dl.nurca	0.07	− 0.08	0.37	0.09	1.7	**94.4**	0.11	− 0.01	0.24	0.08	10.1	**99.4**
L.dl.bedsi	− 0.05	− 0.27	0.04	0.04	21.7	**92.9**	− 0.03	− 0.12	0.25	0.04	10.6	81.7
L.dl.doctca	0.08	− 0.22	0.48	0.11	2.5	89.7	0.04	− 0.13	0.19	0.10	0.0	87.9
L.d.rat	− 0.34	− 1.39	1.02	0.47	11.1	85.9	− 0.67	− 1.30	− 0.21	0.38	45.5	**100.0**
L.d.physh	− 0.03	− 0.08	0.15	0.02	59.3	84.8	− 0.04	− 0.07	0.08	0.01	61.3	**98.7**
L.dl.persh	− 0.01	− 0.10	0.09	0.02	0.0	84.1	− 0.01	− 0.05	0.02	0.01	4.7	**98.0**
L.d.mort	− 0.01	− 0.07	0.03	0.01	5.7	83.7	− 0.01	− 0.04	0.01	0.01	1.6	**92.7**
L.dl.bedsh	0.01	− 0.04	0.21	0.03	1.5	81.0	0.01	− 0.03	0.16	0.02	2.7	66.7
L.d.puhes	0.62	− 12.43	41.35	0.37	8.1	81.0	− 0.16	− 15.02	4.26	0.34	1.0	62.5
L.dl.ta	0.01	− 0.02	0.03	0.01	0.4	79.2	0.01	0.00	0.03	0.01	0.0	**96.2**
L.d.pop65	− 0.53	− 2.99	1.24	1.32	0.0	74.6	0.07	− 2.24	0.99	0.92	0.0	65.3
L.d.texmc	0.53	− 12.38	41.37	0.37	8.0	72.5	− 0.16	− 14.99	4.26	0.31	1.9	69.2
L.d.frp1564	− 0.13	-1.15	0.52	0.29	2.1	72.2	-0.08	− 0.31	0.34	0.26	0.0	73.2
L.d.dp	− 0.20	− 1.11	0.47	0.32	13.0	67.5	− 0.02	− 0.37	0.42	0.25	0.0	70.2
L.dl.gerd	0.00	− 0.07	0.16	0.03	6.4	66.2	− 0.04	− 0.08	0.12	0.03	22.9	**91.7**
L.dl.LE65.F	− 0.06	− 0.86	0.57	0.20	1.1	66.0	0.14	− 0.03	0.50	0.17	0.9	**95.6**
L.dl.accident	0.00	− 0.05	0.02	0.01	1.4	60.3	0.00	− 0.01	0.02	0.01	1.1	67.7
L.d.covero	0.11	− 0.49	3.76	0.28	0.0	58.7	0.11	− 0.56	0.38	0.23	5.0	65.0
L.dl.tobc	0.00	− 0.02	0.01	0.02	0.0	57.7	0.00	− 0.01	0.01	0.01	0.0	52.2
L.dl.alcc	0.00	− 0.10	0.34	0.05	2.0	57.2	0.02	− 0.02	0.15	0.05	0.0	89.9
L.dl.LE65.M	0.02	− 0.36	0.53	0.16	1.3	56.5	0.03	− 0.20	0.30	0.15	0.0	59.3
L.d.ur	− 0.01	− 0.54	0.75	0.27	0.0	51.3	0.07	− 0.24	0.28	0.24	0.0	82.6

OLS-EBA and MM-EBA estimations, with HCE growth as the dependent variable and explanatory variables lagged by one year. For the explanatory variables, the average estimated coefficient (mean *β*), minimum and maximum of the coefficients (min *β* and max *β*), their average standard deviation (Ø se), the proportion of significant coefficients (%Sign.) and the cumulative distribution function CDF(0) of the estimated coefficients are reported. CDF(0) values > 90% and %Sign values > 90% are highlighted in bold

**Table 12:** EBA results for ACE (with year and country FE) using one year lagged explanatory variables

	OLS						MM					
Variables	Mean *β*	Min *β*	Max *β*	Ø se	%Sign	CDF(0)	Mean *β*	Min *β*	Max *β*	Ø se	%Sign	CDF(0)
L.dl.hpi	− 0.12	− 0.25	− 0.02	0.07	39.8	**100.0**	− 0.13	− 0.20	− 0.04	0.06	62.0	**100.0**
L.dl.rend	0.05	− 0.01	0.09	0.02	75.5	**99.9**	0.05	0.03	0.09	0.02	68.3	**100.0**
L.dl.gdp	0.20	− 0.07	0.83	0.11	38.2	**99.8**	0.15	− 0.16	0.51	0.11	18.7	**98.1**
L.dl.sugar	0.03	0.00	0.10	0.02	20.0	**99.7**	0.02	0.00	0.06	0.02	2.3	**99.4**
L.d.pop80	− 3.65	− 13.59	4.82	2.44	27.2	**98.2**	− 2.50	− 7.07	1.25	1.70	26.3	**95.4**
L.dl.Baumol	0.15	− 0.12	0.40	0.08	48.7	**98.0**	0.30	− 0.10	0.51	0.13	59.3	**98.7**
L.d.physh	− 0.02	− 0.07	0.11	0.02	0.0	**96.3**	− 0.02	− 0.06	0.00	0.01	52.5	**100.0**
d.gsh_l1	0.58	− 0.99	2.55	0.52	9.1	**95.7**	0.55	0.05	1.35	0.31	50.3	**100.0**
L.dl.tobc	− 0.01	− 0.02	0.01	0.02	0.0	**92.2**	0.00	− 0.01	0.01	0.01	0.0	**90.7**
L.dl.bedsi	− 0.05	− 0.28	0.04	0.04	20.4	**91.6**	− 0.01	− 0.14	0.03	0.03	1.7	67.2
L.d.puhes	0.65	− 13.12	53.87	0.34	8.8	**90.5**	− 0.26	− 15.14	4.18	0.44	4.0	51.1
L.dl.nurca	0.05	− 0.11	0.38	0.09	2.8	**90.3**	0.09	− 0.04	0.23	0.09	1.1	**93.2**
L.dl.ta	0.01	− 0.02	0.03	0.01	7.0	87.6	0.01	0.00	0.02	0.01	0.0	**97.8**
L.dl.bedsh	0.01	− 0.03	0.22	0.02	2.0	83.8	0.01	− 0.01	0.08	0.02	0.0	**92.1**
L.d.frp1564	− 0.23	− 1.08	0.35	0.27	7.0	83.0	− 0.10	− 0.37	0.26	0.24	0.0	86.8
L.d.texmc	0.52	− 13.11	53.86	0.34	8.3	82.2	− 0.33	− 15.13	4.16	0.50	4.7	58.8
L.d.rat	− 0.34	− 1.53	1.43	0.45	11.3	80.4	− 0.72	− 1.49	− 0.29	0.36	38.6	**100.0**
L.dl.alcc	− 0.02	− 0.10	0.25	0.04	3.1	76.2	0.01	− 0.03	0.13	0.05	0.7	80.1
L.dl.doctca	0.04	− 0.21	0.42	0.10	0.7	75.4	0.08	− 0.11	0.27	0.10	0.0	**93.9**
L.dl.persh	0.00	− 0.07	0.08	0.02	0.0	73.9	− 0.01	− 0.06	0.01	0.01	1.1	**98.9**
L.dl.LE65.F	− 0.07	− 0.86	0.42	0.18	0.9	73.9	0.14	− 0.03	0.45	0.17	5.5	**97.0**
L.d.mort	− 0.01	− 0.06	0.02	0.01	1.2	72.9	− 0.01	− − 0.03	0.01	0.01	3.1	**93.9**
L.d.dp	− 0.19	− 0.95	0.52	0.29	7.7	69.1	− 0.06	− 0.34	0.29	0.23	0.0	77.3
L.dl.accident	0.00	− 0.04	0.02	0.01	1.4	66.9	0.01	− 0.02	0.04	0.01	19.2	**90.8**
L.dl.gerd	0.00	− 0.07	0.17	0.03	6.4	65.5	− 0.04	− 0.08	0.10	0.04	18.8	88.0
L.dl.LE65.M	− 0.02	− 0.43	0.45	0.15	0.1	64.4	0.05	− 0.37	0.38	0.19	0.8	69.7
L.d.covero	0.09	− 1.40	1.82	0.26	0.1	63.0	0.09	− 0.69	0.36	0.16	8.3	56.1
L.d.pop65	− 0.03	− 2.52	1.36	1.23	0.0	52.3	0.63	− 1.31	1.36	0.93	0.0	**92.9**
L.d.ur	0.00	− 0.46	0.87	0.25	0.0	51.9	0.03	− 0.22	0.19	0.25	0.0	71.6

OLS-EBA and MM-EBA estimations, with ACE growth as the dependent variable and explanatory variables lagged by one year. For the explanatory variables, the average estimated coefficient (mean *β*), minimum and maximum of the coefficients (min *β* and max *β*), their average standard deviation (Ø se), the proportion of significant coefficients (%Sign.) and the cumulative distribution function CDF(0) of the estimated coefficients are reported. CDF(0) values > 90% and %Sign values > 90% are highlighted in bold

**Table 13:** EBA results for LTCE (with year and country FE) using one year lagged explanatory variables

	OLS						MM					
Variables	Mean *β*	Min *β*	Max *β*	Ø se	%Sign	CDF(0)	Mean *β*	Min *β*	Max *β*	Ø se	%Sign	CDF(0)
L.d.pop80	− 35.75	− 109.33	7.61	16.04	61.2	**99.9**	− 0.88	− 8.92	5.32	2.95	5.2	54.1
L.dl.alcc	0.78	− 0.27	1.91	0.27	55.7	**99.8**	0.05	− 0.29	0.23	0.09	1.0	**93.8**
L.dl.doctca	1.88	− 0.47	6.32	0.65	76.5	**98.8**	− 0.16	− 0.32	0.20	0.16	12.3	84.5
L.dl.persh	− 0.11	− 0.66	0.09	0.09	8.9	**98.5**	− 0.03	− 0.20	0.27	0.03	10.8	**96.9**
L.d.mort	− 0.10	− 0.50	0.08	0.08	7.7	**97.5**	− 0.02	− 0.06	0.02	0.02	8.8	**95.6**
L.d.texmc	0.82	− 72.00	17.15	2.28	0.5	**91.3**	− 0.15	− 6.14	8.07	0.55	2.6	85.6
L.d.rat	− 1.93	− 8.03	3.53	2.80	1.3	**90.4**	− 0.04	− 0.90	0.70	0.75	0.7	57.4
L.dl.accident	0.04	− 0.09	0.12	0.07	0.0	88.8	− 0.01	− 0.04	0.01	0.02	0.0	**94.1**
L.dl.Baumol	0.28	− 0.63	1.03	0.51	4.2	88.7	0.37	− 0.01	0.76	0.16	68.7	**99.9**
L.d.puhes	− 0.05	− 72.15	16.65	2.28	0.2	85.7	− 0.22	− 6.19	8.02	0.56	2.1	**93.7**
L.dl.bedsi	− 0.47	− 3.01	0.52	0.27	34.5	82.3	-0.07	− 0.36	− 0.01	0.06	7.0	**100.0**
L.dl.tobc	0.02	− 0.06	0.08	0.06	0.0	80.8	0.00	− 0.02	0.02	0.02	0.0	56.7
L.dl.gdp	0.77	− 1.39	3.16	0.69	16.8	80.6	− 0.17	− 0.49	0.42	0.20	8.2	87.6
L.d.dp	0.64	− 1.33	4.39	2.01	0.4	79.3	0.34	− 0.22	1.06	0.39	4.7	**92.5**
L.dl.LE65.M	0.12	− 2.74	1.46	0.98	0.1	74.5	0.29	− 0.03	0.66	0.24	3.0	**99.5**
L.d.covero	0.73	− 1.10	23.98	1.06	0.2	73.6	0.13	− 0.75	2.39	0.24	5.3	60.8
L.dl.LE65.F	− 0.40	− 4.59	1.92	1.20	1.6	67.3	0.40	− 0.02	0.81	0.23	52.6	**99.5**
L.d.frp1564	− 2.22	− 8.85	2.04	1.77	25.9	66.4	0.29	− 0.29	0.90	0.37	0.5	**97.3**
L.d.pop65	2.78	− 12.48	21.52	8.11	0.6	65.1	− 2.79	− 4.94	− 0.88	1.44	51.8	**100.0**
L.dl.bedsh	0.10	− 0.36	0.80	0.18	0.3	64.5	− 0.03	− 0.14	0.10	0.03	4.6	**96.4**
L.dl.nurca	− 0.10	− 0.84	1.23	0.58	0.6	63.0	0.07	− 0.08	0.31	0.09	0.0	**95.5**
L.dl.ta	0.01	− 0.17	0.12	0.08	0.1	61.7	0.01	− 0.11	0.02	0.01	0.5	81.9
L.d.physh	− 0.04	− 0.29	0.58	0.09	21.5	61.3	− 0.08	− 0.35	0.22	0.04	52.8	53.8
L.d.ur	− 0.38	− 2.87	2.14	1.67	1.6	60.4	0.32	− 0.60	1.28	0.36	2.0	88.8
d.gsh_l1	− 1.18	− 15.19	4.22	3.41	0.0	59.3	0.78	− 0.12	1.39	0.44	65.3	**99.0**
L.dl.gerd	− 0.03	− 0.39	0.51	0.23	0.3	58.9	− 0.01	− 0.08	0.20	0.06	0.4	78.8
L.dl.rend	− 0.02	− 0.30	0.11	0.09	0.1	56.1	0.03	− 0.10	0.09	0.04	40.3	88.1
L.dl.hpi	0.04	− 1.18	1.82	0.52	6.8	55.2	− 0.11	− 0.27	0.09	0.15	0.0	**91.8**
L.dl.sugar	− 0.06	− 0.42	0.74	0.14	23.8	53.2	0.02	− 0.05	0.06	0.03	0.6	**93.2**

OLS-EBA and MM-EBA estimations, with LTCE growth as the dependent variable and explanatory variables lagged by one year. For the explanatory variables, the average estimated coefficient (mean *β*), minimum and maximum of the coefficients (min *β* and max *β*), their average standard deviation (Ø se), the proportion of significant coefficients (%Sign.) and the cumulative distribution function CDF(0) of the estimated coefficients are reported. CDF(0) values > 90% and %Sign values > 90% are highlighted in bold

## Footnotes

1 The term ‘Baumol’s cost disease’ was coined by Alice Vandermeulen (1968), see Baumol (2012, p. xii).

2 See also Lorenzoni et al. (2019, p. 40) for a single-page derivation of Hartwig’s cost disease variable.

3 The growth rate of aggregate RULC equals the growth rate of the wage share in GDP.

4 Helland and Tabarrok (2019) prefer the expression ‘Baumol effect’ since it avoids the negative connotations of the term ‘Baumol’s (cost) disease’.

5 Rossen and Faroque (2016, p. 203), who only perform the nominal version of the test, conclude that “Baumol’s cost disease on health-care spending increases in Canada may not be economically very important”. Bates and Santerre (2013) and Wang and Chen (2021) also find relatively minor effects. The main reason for this finding, however, seems to be that these authors apply a ‘correction’ to Hartwig’s ‘Baumol variable’ suggested by Colombier (2012). The main effect of this ‘correction’ is that it scales the estimated coefficient down by a factor around 10 (see Table 2 in Rossen and Faroque 2016).

6 See https://en.wikipedia.org/wiki/Acute_care.

7 We mostly used the 2020 version of the OECD Health database. All data are available on request to the corresponding author.

8 Hartwig and Sturm’s data were also used by Hauck and Zhang (2016).

9 We found no sources to update the dummy variables used by Hartwig and Sturm (2014) to model the institutional specifics of the national health systems. Since none of these institutional dummy variables turned out as a robust and statistical significant explanatory variable for HCE growth in Hartwig and Sturm’s EBAs, and in order not to forego the most recent years by including the non-updated dummies, we decided to drop them from our analysis.

10 For technical reasons, the function *lmrob* cannot calculate MM estimates with country-specific trends.

11 Parts of this section rely upon previous work (Hartwig and Sturm 2014).

12 Criticism of EBA can be found in McAleer et al. (1985), Breusch (1990), and Angrist and Pischke (2010).

13 Hauck and Zhang (2016) use Bayesian Model Averaging to identify robust drivers of HCE growth. They work around the problem of missing observations by imputing missing values.

14 Increasing Gaussian efficiency would lead to a higher estimation bias making comparison between the OLS and MM-regression based results more difficult.

15 The number N of sub-samples to generate is chosen to guarantee that at least one subset without outliers is selected with high probability (see Salibian-Barrera and Yohai 2006, on how to achieve this).

16 Using the function *lmrob* in R, we apply the S-estimator proposed by Rousseeuw and Yohai (1984) as initial estimator.

17 We lagged the variable ‘(change in the) government share (d.gsh)’ in the baseline version of our EBAs already due to obvious endogeneity concerns with respect to this variable.

18 For example, the sign on the growth rate of the health price index (dl.hpi) is negative in 99.1% of the OLS regressions, which is counter-intuitive, and the variable is significant in 63.1% of them. We mentioned in the introduction that health price indices are unreliable.

19 For the Baumol coefficients estimated with OLS: (2) $t=\frac{{\mu }_{ACE}-{\mu }_{HCE}}{\sqrt{\frac{{\sigma }_{ACE}^{2}}{{n}_{ACE}}+\frac{{\sigma }_{HCE}^{2}}{{n}_{HCE}}}}=\frac{0.642-0.688}{\sqrt{\frac{{0.0500}^{2}}{20853}+\frac{{0.0496}^{2}}{20853}}}=-94.78$, while for Baumol coefficients estimated with MM: (3) $t=\frac{0.693-0.720}{\sqrt{\frac{{0.0407}^{2}}{1179}+\frac{{0.0385}^{2}}{1408}}}=-16.61$.

20 In the case of the Baumol coefficients estimated with OLS, we obtain: (4) $t=\frac{{\mu }_{ACE}-{\mu }_{LTCE}}{\sqrt{\frac{{\sigma }_{ACE}^{2}}{{n}_{ACE}}+\frac{{\sigma }_{LTCE}^{2}}{{n}_{LTCE}}}}=\frac{0.642-0.787}{\sqrt{\frac{{0.0500}^{2}}{20853}+\frac{{0.2559}^{2}}{20853}}}=-80.39$, whereas for the Baumol coefficients estimated with MM we get: (5) $t=\frac{0.693-0.923}{\sqrt{\frac{{0.0407}^{2}}{1179}+\frac{{0.1126}^{2}}{1972}}}=-81.92$.

21 This result puts into perspective our earlier finding based on a seemingly unrelated regression that the impact of the Baumol variable on ACE is not different from its impact on LTCE (see Table 6).

22 The impact of unemployment on LTCE growth is much higher than on ACE growth.

23 That the coefficient on dl.doctca is negative on average may seem implausible given that ‘too many doctors’ are thought to increase health costs. This seems not to be the case in long-term care, though.

24 Revisiting the question whether the Baumol effect affects ACE and LTCE differently, these findings reinforce the conclusion that the effect on LTCE is stronger.
